# Antibiofilm Property and Biocompatibility of Siloxane-Based Polymer Coatings Applied to Biomaterials

**DOI:** 10.3390/ma16237399

**Published:** 2023-11-28

**Authors:** Akiko Ogawa, Akane Tahori, Mayumi Yano, Shunma Hirobe, Satoshi Terada, Hideyuki Kanematsu

**Affiliations:** 1 Department of Chemistry and Biochemistry, National Institute of Technology (KOSEN), Suzuka College, Suzuka 510-0294, Japan; akane.tahochan@gmail.com (A.T.); my.storm.1106@gmail.com (M.Y.); 2 Faculty of Engineering, Division of Engineering Applied Chemistry and Biochemistry, University of Fukui, Fukui 910-0017, Japan; syokaku99@gmail.com (S.H.); terada@u-fukui.ac.jp (S.T.); 3 Department of Materials Science and Engineering, National Institute of Technology (KOSEN), Suzuka College, Suzuka 510-0294, Japan; kanemats@suzuka.kosen-ac.jp

**Keywords:** biofilm, infectious diseases, biomaterials, siloxane-based polymer coating, crystal-violet staining, cytotoxicity

## Abstract

Biofilm infections sometimes occur on biomaterials inserted into the body because biomaterials can block the attack of immune cells such as macrophages, promoting biofilm formation by invading bacteria. Owing to their use in antifouling applications, including biofilm formation, siloxane-based polymer coatings are considered a promising method to prevent biofilm formation on the surface of biomaterials. In this study, we explored the antibiofilm property and biocompatibility of siloxane-based polymer coatings. Biofilm formation and cytotoxicity tests were performed using *Escherichia coli* and *Staphylococcus epidermidis* to quantify the biofilms while U937 cells were used to measure the time course of viable cell concentration and viability, respectively. In both the biofilm formation and cytotoxicity tests, stainless steel SUS316L plates and titanium plates coated with the siloxane-based polymer and sterilized in an autoclave were used as the biomaterials. The amount of biofilm formed on the polymer-coated titanium plate was substantially higher than that on a noncoated titanium plate in the case of *S. epidermidis*. The viable cell concentration and viability of U937 cultured on the polymer-coated titanium plate were lower than those of U937 cultured on the noncoated titanium plate. The same trend was observed between polymer-coated and noncoated SUS316L plates. These results indicate that the siloxane-based polymer coatings need additional treatment to achieve a satisfactory antibiofilm property and that they are sensitive to autoclave treatment, resulting in cytotoxicity.

## 1. Introduction

Biofilm-related infections are caused by pathogenic bacteria that form biofilms in the human body. The internal bacteria in biofilms are embedded in the extracellular polymeric substance (EPS) matrix produced by themselves and exhibit higher survival rates than planktonic bacteria because of antibiotic tolerance, resulting in chronic and multiple infections [1]. Biofilm-related infections pose serious risks to patients with immunity disorders, those using inserted medical devices, and those being treated for chronic diseases.

Biofilm formation proceeds in the following four stages: (1) First, conditioning films are formed by inorganic and organic compounds derived from blood and bodily fluids on the surface of medical devices. (2) Invading pathogenic bacteria irreversibly attach and then grow on the conditioning films. (3) Aggregated bacteria multiply, thus producing the EPS matrix, which results in the maturation of the biofilms with the aggregated bacteria embedded in the EPS matrix. (4) Matured biofilms partially break up, and the suspended parts flow with blood or bodily fluids until the mixture of EPS and pathogenic bacteria find another suitable growing area and attach again [2].

Inserted medical devices such as catheters used to open urinary tract obstruction, artificial joints, and stents used to expand valves and vascular tracts are made of polymers and metallic alloys, which are usually human-friendly materials. However, these devices can accelerate biofilm formation because immune cells such as macrophages in patients cannot pass through these devices and cannot attack the biofilm-forming pathogenic bacteria. Once biofilms are formed on the artificial devices, removing the biofilms is very difficult due to their antibiotic tolerance. Therefore, the regulation of biofilm formation in early stages (stages 1–2) is the key point to prevent severe infectious diseases in patients.

To fight against biofilm-related infections occurring with inserted materials, modification of the material surface by using coatings having antibacterial and antibiofilm properties constitutes an attractive strategy. We previously investigated the application of siloxane-based polymer coatings that had been studied by D&D Co. (Yokkaichi, Japan) for the prevention of biofilm formation in medical devices. Siloxane-based polymer coatings, which consist of a siloxane backbone as the main chain and methyl and phenyl groups as side chains [3], contain free spaces that can accommodate nanosized particles such as antibiofilm effective metals and capsulized medicines, thus prolonging the inhibitory effects of these compounds on biofilm formation. In our study, siloxane-based polymer coatings containing dispersed silver or copper nanoparticles were found to considerably inhibit biofilm formation in a cooling water system [4].

**Figure 1:**
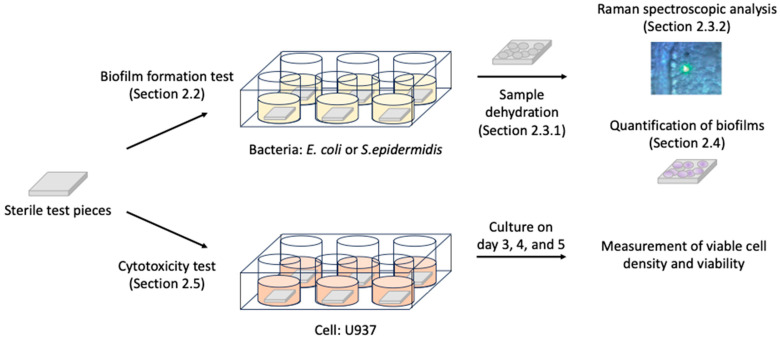
Outline of the experimental procedure. Parenthesized words indicate the relevant sections describing the processes in detail in Section 2.

For applying siloxane-based polymer coatings in the medical field, the biological safety of the coatings and the influence of the sterilizing process on the features of the coatings must be considered. However, these characteristics have not been evaluated yet. Herein, we report the investigation of biofilm formation and biocompatibility of siloxane-based polymers. Based on the assumption that siloxane-based polymers are used as biomaterials, SUS316L plates and pure titanium plates were used as the basal materials that were covered with siloxane-based polymers. *Staphylococcus epidermidis* (Gram-positive bacteria) and *Escherichia coli* (Gram-negative bacteria) were used as model bacteria to evaluate quantitatively the biofilm formation assay, and a human monocyte cell line, U937, was used for the cytotoxicity assay (Figure 1).

## 2. Materials and Methods

### 2.1. Specimens

SUS316L stainless steel plates (thickness: 1 mm) and pure titanium plates (pure grade: 99.4%; thickness: 1 mm) were purchased from Nilaco Co. (Tokyo, Japan) and AS ONE Co. (Osaka, Japan), respectively. These were cut into squares of 10 mm × 10 mm. Each test piece was washed with acetone and stored in a desiccator (AS ONE) for 24 h. Next, all the test pieces were coated with the siloxane-based polymer. The coating was prepared by Dr. Katsuhiko Sano (D&D Co., Yokkaichi, Japan) as described in Section 2.1.1.

#### 2.1.1. Preparation of Siloxane-Based Polymer Coating

1. An alkylalkoxysilane permeate having methyl and phenyl groups (8.0 g, D&D Co.) was mixed with *N*-2-(aminomethyl)-3-aminopropyltrimethoxysilane (2.0 g, KBM-603, Shin-Etsu Chemical Co., Tokyo, Japan) in a 100 mL polypropylene cup at 1000 rpm for 10 min using a stirring device (PRIMIX, Tokyo, Japan).
2. The mixture was filtered with a #110 nylon mesh (AS ONE Co.).
3. The filtered mixture was packed into an air-spray gun (Airtex, Osaka, Japan).
4. The coating reagent (from (3)) was applied to the clean test pieces until the thickness of the coating was about 10 μm.
5. After application, the coated test pieces were allowed to cure at 18–25 °C for seven days.

#### 2.1.2. Sterilization of Specimens

An autoclave was used for sterilization. Before sterilization, all specimens were wiped ten times on both sides using a wiper containing 70% ethanol and then stored in sterile glass beakers (AGC Co., Tokyo, Japan). The autoclave treatment was performed at 121 °C for 15 min.

### 2.2. Biofilm Formation Test

*Staphylococcus epidermidis* (ATCC number: 35984, ATCC, Manassas, VA, USA) and *Escherichia coli* K-12 strain (JCM No. 20135, RIKEN BRC, Tsukuba, Japan) were used as Gram-negative and Gram-negative model bacteria, respectively. These bacteria were stored on Microbank beads (Iwaki Co., Tokyo, Japan) at −20 °C. One Microbank bead was added to a test tube containing 5 mL of culture medium, i.e., LB medium Lennox (Nacalai Tesque, Inc., Kyoto, Japan) for *E. coli* K-12 and Tryptic Soy Broth (BD, Franklin Lakes, NJ, USA) for *S. epidermidis*, and incubated at 37 °C for 16 h with shaking at 100 rpm (TAITEC, Koshigaya, Japan). Each sterile test sample was placed in a well of a six-well culture plate (AS ONE) containing 3 mL of culture medium and 2 mL of culture solution. *E. coli* K-12 was cultured at 25 °C for 24 h, and *S. epidermidis* was cultured at 37 °C for 24 h.

### 2.3. Sample Preparation for Raman Spectroscopic Analysis

After the culture test described in Section 2.2, each specimen was taken out from the culture solution and transferred to an acrylic sample case (AS ONE) with the biofilm-adhered side facing up. All samples were dehydrated according to the procedure described in Section 2.3.1 before Raman spectroscopic analysis.

#### 2.3.1. Sample Dehydration

1. One mL of 30% (*v*/*v*) ethanol was added to the sample case and left for 15 min, then aspirated out using a micropipette.
2. The same procedure as in step 2 was repeated with 50% (*v*/*v*), 60% (*v*/*v*), 70% (*v*/*v*), 80% (*v*/*v*), 90% (*v*/*v*), 95% (*v*/*v*), 98% (*v*/*v*), and 99.5% (*v*/*v*) ethanol followed by 30% (*v*/*v*) t-butanol/70% (*v*/*v*) ethanol, 50% (*v*/*v*) t-butanol/50% (*v*/*v*) ethanol, 70% (*v*/*v*) t-butanol/30% (*v*/*v*) ethanol, and t-butanol, in that order.
3. The samples were left in a refrigerator (5–10 °C) for 30 min.
4. The sample cases were placed in a desiccator (AS ONE) with the lids open, connected to an aspirator (AS ONE), and aspirated for 60 min to dry the solvent completely.
5. The dehydrated samples were stored in desiccators (AS ONE) until the Raman spectroscopic observation was performed.

#### 2.3.2. Raman Spectroscopic Analysis

Each sample was randomly observed at five points using the supplemental microscope (100× magnitude) of a Raman spectroscopic analyzer (JASCO Co., Tokyo, Japan) and photographed, and the Raman spectrum was then measured with a wavenumber range of 500–4000 cm^−1^, an exposure time of 0.3 s, a scanning number of two, and a slit width of 0.1 mm × 6 mm.

### 2.4. Quantification of Biofilms

After the Raman spectroscopic observation, each dehydrated sample was immersed in a 50 mL centrifuge tube containing 15 mL of crystal-violet solution (0.1 wt.%) for 30 min at 25 °C. The stained samples were removed from the solution, washed with running tap water in a stainless container for 3 min, and dried on a paper towel for 10 min. Scotch tape was applied to the top side of the sample and scratched over the tape ten times with the belly of the finger. The tape was then removed and attached to a glass slide. The L*a*b* color space was measured three times per sample using a colorimeter (Konica Minolta Inc., Tokyo, Japan) from the opposite side of the Scotch-tape-attached slide glass. The amount of biofilm was calculated using Equation (1).

(1) $$\mathrm{Amount}\;\mathrm{of}\;\mathrm{biofilm}=\mathrm{square}\;\mathrm{root}\;\left\{{\left(100-L^{*}\right)}^{2}+{\left(a^{*}\right)}^{2}+{\left(b^{*}\right)}^{2}\right\}$$

### 2.5. Cytotoxicity Test

U937 cells that were kindly gifted by Professor Hidekazu Tamauchi (Daito Bunka University) were maintained in RPMI 1640 medium (Shimadzu Diagnostics Co., Tokyo, Japan) containing 5% fetal bovine serum (Thermo Fisher Scientifics, Walthan, MA, USA), 4 mM glutamine (Wako-Fujifilm, Osaka, Japan), 0.2% sodium hydrogen carbonate (Wako-Fujifilm), and 5 mM HEPES (Thermo Fisher Scientifics). Each sterile test piece was put into a well of a six-well culture plate (Sumitomo Bakelite Co., Ltd., Tokyo, Japan), and subconfluent U937 cells were seeded at 3.31 × 10^4^ cells/mL (*n* = 3). These were cultured in a CO_2_ incubator (Panasonic, Osaka, Japan) at 36.5 °C, 5% CO_2_, and 99% humidity for five days. On days 3, 4, and 5 of the culture, each well was pipetted four times to collect 100 μL of culture solution, and the viable cell concentration and viability were determined using the trypan blue exclusion method and by counting the cell number with a hemocytometer under a microscope.

### 2.6. Statistical Analysis

Welch’s *t*-test was used for statistical analysis to compare the average value between the control condition and the target condition.

## 3. Results

Four samples were prepared for the biofilm formation and cytotoxicity tests, i.e., pure titanium and stainless steel SUS316L, which are hereinafter referred to as Ti and 316L, respectively, and siloxane-based-polymer-coated pure titanium and siloxane-based-polymer-coated stainless steel SUS316L, hereinafter referred to as Coated-Ti and Coated-316L, respectively. Sterile samples were placed on the culture-side bottom of the wells and submerged in the culture solution during the culture period. The name of the submerged samples indicates both the culture condition and the specimen name, that is, before-culture Coated-Ti, after-culture Coated-Ti, before-culture Coated-316L, and after-culture Coated-316L.

### 3.1. Biofilm Formation Test 

#### 3.1.1. Biofilm Formation Test in *E. coli*

**Figure 2:**
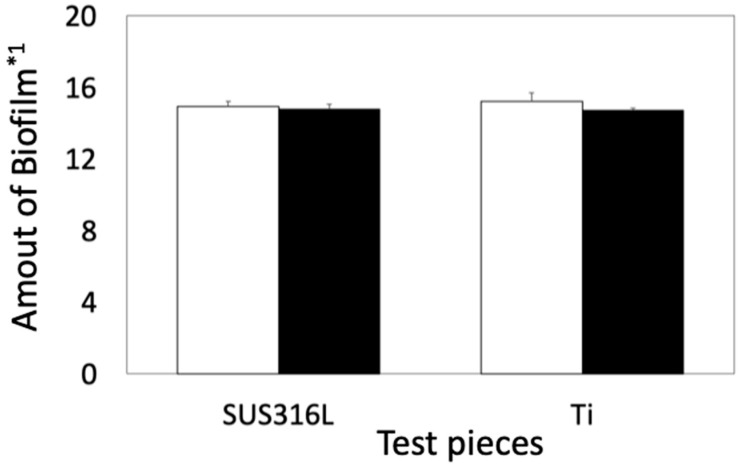
Quantitative comparison of biofilms between noncoated and coated samples in *E. coli*. Open and solid bars indicate the amount of biofilms formed on the basal plates and on the siloxane-based-polymer-coated basal plates, respectively. *1: The amount of biofilm was calculated using Equation (1). Error bars indicate the standard deviation (*n* = 3).

The amount of biofilm was very similar among the four culture conditions (Figure 2).

**Figure 3:**
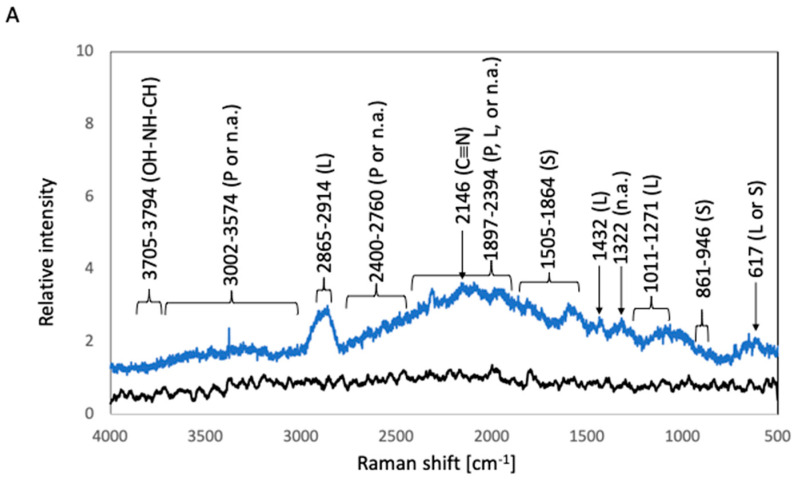

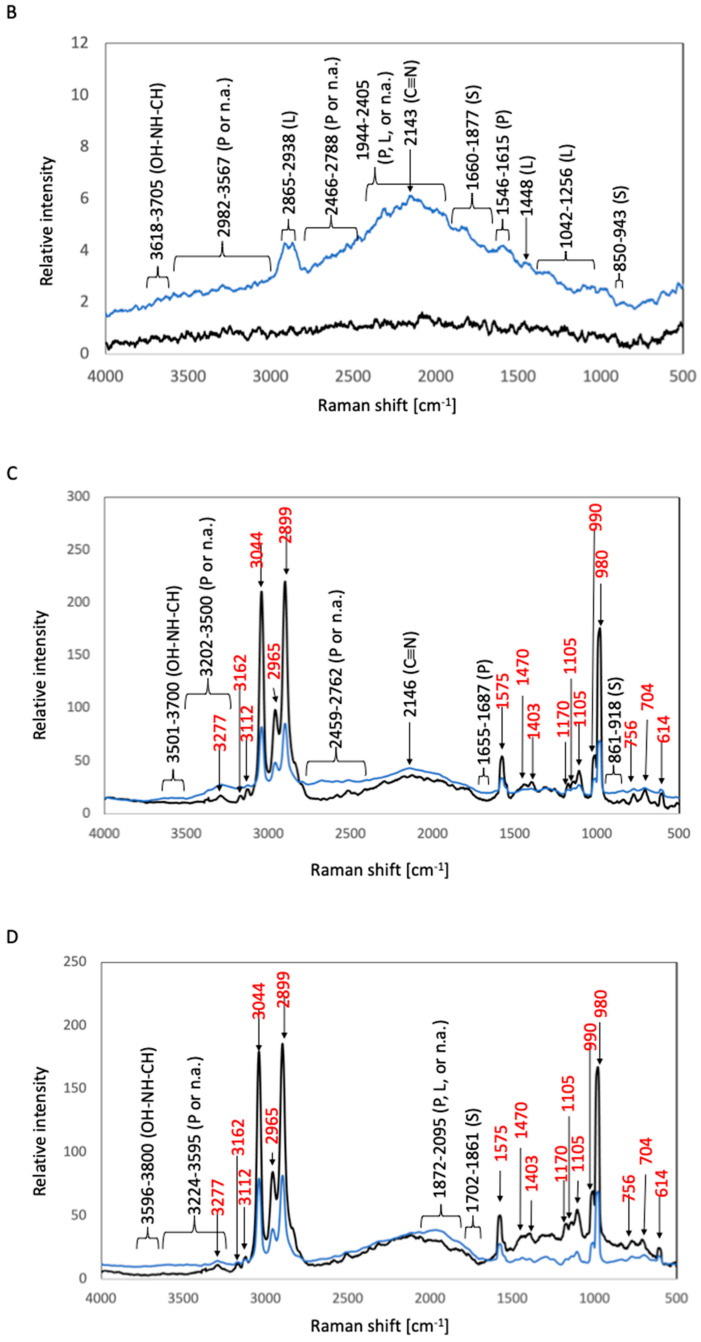
Raman spectra of biofilms in *E. coli* cultures. The black and blue lines represent the Raman peaks of the samples before and after *E. coli* culture, respectively. (**A**) 316L; (**B**) Ti; (**C**) Coated-316L; (**D**) Coated-Ti. Abbreviations: P—proteins; L—lipids; S—polysaccharides; n.a.—nucleic acids. The red numbers indicate Raman shifts associated with the siloxane-based polymer.

To estimate the components of the biofilms, the sample surfaces were analyzed by Raman spectroscopy. The relative intensities of the Raman shifts are summarized in Figure 3. The surfaces of before-culture SUS316L and Ti were covered with oxidized layers. E.J. Ekoi et al. reported that two major peaks were detected at 447 cm^−1^ and 610 cm^−1^ for titanium oxide (TiO_2_) [5]. Y. Matsuda et al. reported that strong Raman peaks were detected at 554 cm^−1^ for chromium oxide (Cr_2_O_3_), 680 cm^−1^ for manganese oxide (MnCr_2_O_4_), and 681 cm^−1^ for iron oxide (FeCr_2_O_4_) [6]. Based on this information, Raman shift peaks derived from such oxidized layers should be detected in the before-culture SUS316L and Ti plates. However, these Raman shift positions were covered by background noise, therefore, the Raman spectra of before-culture SUS316L and Ti were treated as baselines. The main components of the biofilms were deduced from the position of particular Raman peaks according to previous reports [7,8,9,10,11,12,13,14,15,16,17]. In the case of after-culture 316L, the highest Raman peak, which was detected at 2146 cm^−1^, can be attributed to C≡N triple-bond stretching [7]. Lipid-associated peaks were detected at 1011–1271 cm^−1^ (C–O–O stretching vibrations), 1432 cm^−1^ (hydrocarbon chain, –CH_2_ scissoring, and twisting vibrations), and 2865–2914 cm^−1^ (C–H stretching modes) [8,9]. A nucleic-acid-associated peak and polysaccharide-associated peaks were detected at 1322 cm^−1^ (P=O-containing compounds) and 1505–1741 cm^−1^ (attributed to carbonyl compounds), respectively [8]. A peak observed at 617 cm^−1^ was related to polysaccharides or lipids [8]. The peaks between 1897 and 2394 cm^−1^ can be ascribed to the vibration of C=C or C=O bonds of fatty acids, amide bonds of proteins, and DNA-strand bonds [9,10,11]. Bond vibrations of proteins or nucleic acids resulted in broad peaks at 2400–2760 and 3002–3574 cm^−1^ [12]. Broad peaks attributable to OH–NH–CH stretching vibration were observed at 3705–3794 cm^−1^ [13]. In the case of after-culture Ti, the highest Raman peak, which was related to C≡N triple-bond stretching, appeared at 2143 cm^−1^ [7]. Lipid-associated peaks were detected at 1042–1256 cm^−1^ (C–O–O stretching vibration), 1448 cm^−1^ (hydrocarbon chain, –CH_2_ scissoring, and twisting vibrations), and 2865–2938 cm^−1^ (C–H stretching modes) [8,9].Protein-related peaks appeared at 1546–1615 cm^−1^ (C=O stretching vibration of peptide linkages) [8]. Polysaccharide-related peaks were detected at 850–943 cm^−1^ (related to carbohydrates) and 1660–1877 cm^−1^ (attributed to carbonyl compounds) [14]. The vibration of C=C or C=O bonds of fatty acids, amide bonds of proteins, and DNA-strand bonds produced peaks at 1944–2405 cm^−1^ [9,10,11]. Meanwhile, bond vibrations of proteins or nucleic acids afforded broad peaks at 2865–2938 and 2982–3567 cm^−1^ [12]. A peak due to the OH–NH–CH stretching vibration was detected at 3618–3705 cm^−1^ [7].

Before-culture Coated-316L and before-culture Coated-Ti gave rise to sharp peaks at 614, 704, 756, 980, 990, 1105, 1114, 1170, 1403, 1447, 1575, 2899, 2965, 3044, 3112, 3162, and 3277 cm^−1^. These peaks were also detected in after-culture Coated-316L and after-culture Coated-Ti; therefore, they can be attributed to chemical bonds related to the siloxane-based polymer. In particular, the peaks at 980 and 1105 cm^−1^ were identified as characteristic peaks of the Si–O stretching vibration and Si–O–Si antisymmetric stretching vibration, respectively [15]. In the case of after-culture Coated-316L, a peak due to C≡N triple-bond stretching was detected at 2145 cm^−1^ [7].Polysaccharide-related broad peaks were observed at 861–946 cm^−1^ (the skeleton mode of the anomeric skeletal configuration), and protein-related broad peaks were detected at 1655–1687 cm^−1^ (C=O stretching vibration of peptide linkages) [8]. Bond vibrations of proteins or nucleic acids afforded broad peaks at 2459–2762 and 3202–3500 cm^−1^ [12]. The stretching vibration peak of OH–NH–CH appeared at 3501–3700 cm^−1^ [13]. In the case of after-culture Coated-Ti, polysaccharide-related broad peaks were detected at 1702–1861 cm^−1^ (attributed to carbonyl compounds) [14]. The vibration of C=C or C=O bonds of fatty acids, amide bonds of proteins, and DNA-strand bonds afforded peaks at 1872–2095 cm^−1^ [9,10,11]. Bond vibrations of proteins or nucleic acids gave rise to broad peaks at 3224–3595 cm^−1^ [12]. Broad peaks derived from the stretching vibration of OH–NH–CH were detected at 3596–3800 cm^−1^ [7].

#### 3.1.2. Biofilm Formation Test in *S. epidermidis*

**Figure 4:**
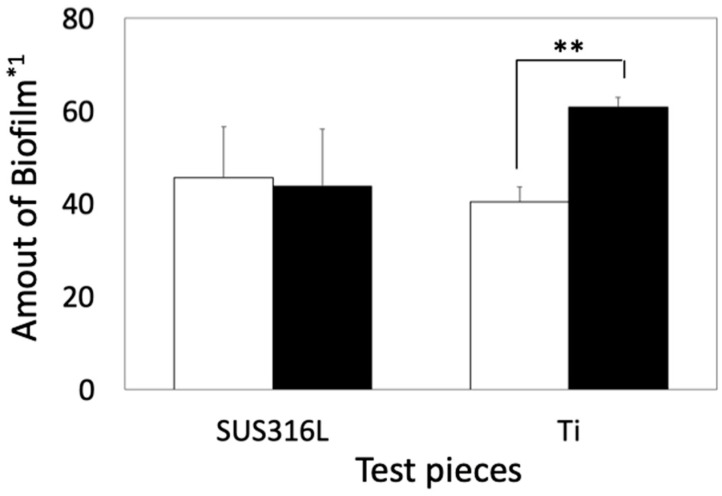
Quantitative comparison of biofilms between noncoated and coated samples in *S. epidermidis*. Open and solid bars indicate the amount of biofilms formed on the basal plates and siloxane-based-polymer-coated basal plates, respectively. *1: The amount of biofilm was calculated using Equation (1). Error bars indicate standard deviation (*n* = 3). **: *p* < 0.01.

The amount of biofilm formed in Coated-Ti was substantially higher than that in Ti. In contrast, the amount of biofilm formed in Coated-316L was very similar to that in 316L (Figure 4).

**Figure 5:**
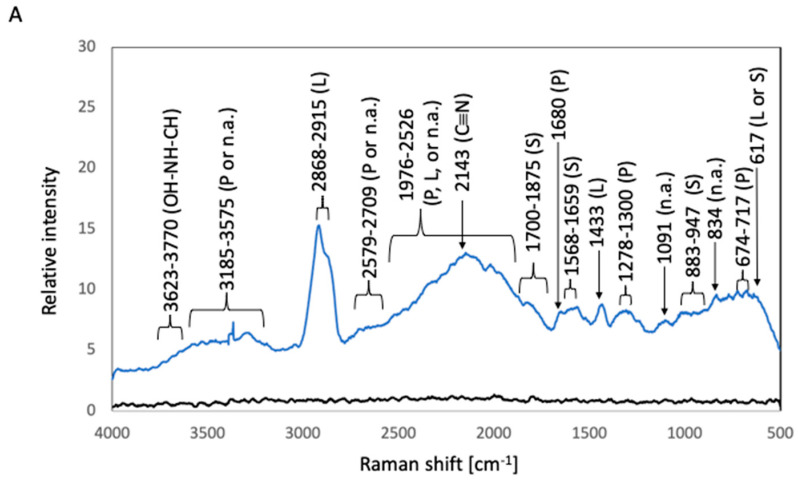

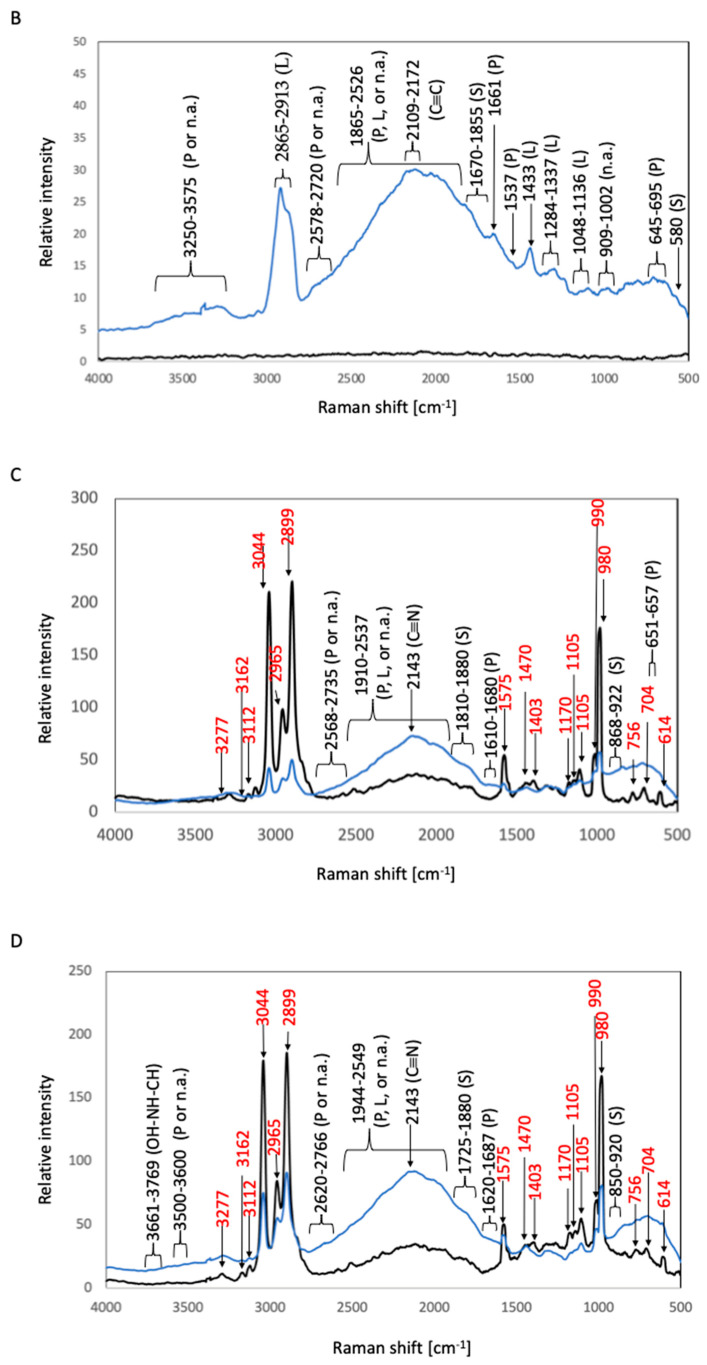
Raman spectra of biofilms in *S. epidermidis* cultures. The black and blue lines represent the Raman peaks of the samples before and after *S. epidermidis* culture, respectively. (**A**) 316L; (**B**) Ti; (**C**) Coated-316L; (**D**) Coated-Ti. Abbreviations: P—proteins; L—lipids; S—polysaccharides; n.a.—nucleic acids. The red numbers indicate Raman shifts associated with the siloxane-based polymer.

The corresponding Raman shifts are summarized in Figure 5. In the spectrum of after-culture 316L, the highest Raman peak, which was detected at 2868–2915 cm^−1^, was related to the C–H stretching modes of lipids [9]. A peak at 617 cm^−1^ can be attributed to polysaccharides or lipids [8,14]. A lipid-related peak was observed at 1433 cm^−1^ (hydrocarbon chain, –CH_2_ scissoring, and twisting vibrations) [9]. Protein-related peaks were detected at 674–717 cm^−1^ (a mixture of C–S stretching with H, C–S stretching with C, and CH wagging vibration), 1278–1300 cm^−1^, and 1680 cm^−1^ (C=O stretching vibration of peptide linkages) [8,16]. Polysaccharide-related peaks were observed at 883–947 cm^−1^ (the skeleton mode of the anomeric skeletal configuration) and at 1568–1659 and 1700–1875 cm^−1^ (attributed to carbonyl compounds) [12]. Nucleic-acid-related peaks were detected at 834 and 1091 cm^−1^ (DNA backbone). The vibration of C=C or C=O bonds of fatty acids, amide bonds of proteins, and DNA-strand bonds afforded peaks at 1876–2526 cm^−1^ [8,14]. A C≡N triple-bond stretching peak appeared at 2143 cm^−1^ [7]. Bond vibrations of proteins or nucleic acids resulted in broad beaks at 2579–2709 and 3185–3575 cm^−1^ [12]. Broad peaks attributable to the OH–NH–CH stretching vibration were detected at 3623–3770 cm^−1^ [7]. In the case of after-culture Ti, the highest Raman peak was detected at 2109–2172 cm^−1^ and was related to the C≡C triple-bond stretching vibration [5]. Lipid-associated peaks were observed at 1048–1136 cm^−1^ (C–C stretching vibration of fatty acids), 1284–1337 cm^−1^ (HC=CH stretching vibration of unsaturated fatty acids), 1433 cm^−1^ (hydrocarbon chain, –CH_2_ scissoring, and twisting vibrations), and 2865–2913 cm^−1^ (C–H stretching modes) [6,7]. Protein-related peaks were detected at 645–696 cm^−1^ (C–S stretching and C–C twisting of proteins) and at 1537 and 1661 cm^−1^ (C=O stretching vibration of peptide linkages) [8,17]. Polysaccharide-related peaks were observed at 580 cm^−1^ (related to carbohydrates) and 1670–1855 cm^−1^ (attributed to carbonyl compounds) [14]. Nucleic-acid-related peaks were detected at 909–1002 cm^−1^ (P–O stretching) [8]. The vibration of C=C or C=O bonds of fatty acids, amide bonds of proteins, and DNA-strand bonds gave rise to peaks at 1865–2526 cm^−1^ [9,10,11]. Broad peaks due to bond vibrations of proteins or nucleic acids appeared at 2578–2720 and 3250–3575 cm^−1^ [12].

In the Raman spectra of before-culture Coated-Ti, before-culture Coated-316L, after-culture Coated-Ti, and after-culture Coated-316L, sharp peaks ascribable to the chemical bonds of the siloxane-based polymer were detected at 614, 704, 760, 980, 990, 1105, 1575, 2899, 2965, and 3044 cm^−1^, as found in the test with *E. coli* (Section 3.1.1.). In the spectrum of after-culture Coated-316L, the highest Raman peak was observed at 2143 cm^−1^ and corresponded to the C≡N triple-bond stretching [7]. Protein-related peaks were detected at 657–661 cm^−1^ (C–S stretching and C–C twisting of proteins) and 1610–1680 cm^−1^ (C=O stretching vibration of peptide linkages) [8,17]. Polysaccharide-related peaks appeared at 868–922 cm^−1^ (the skeleton mode of the anomeric skeletal configuration) and 1810–1880 cm^−1^ (attributed to carbonyl compounds) [14]. The vibration of C=C or C=O bonds of fatty acids, amide bonds of proteins, and DNA-strand bonds gave rise to peaks at 1910–2537 cm^−1^ [9,10,11]. Broad beaks appearing at 2568–2735 cm^−1^ can be ascribed to the bond vibration of proteins or nucleic acids [12]. In the case of after-culture Coated-Ti, the highest Raman peak, which was detected at 2143 cm^−1^, was related to the C≡N triple-bond stretching [7]. Protein-related peaks were observed at 1620–1687 cm^−1^ (C=O stretching vibration of peptide linkages) [8]. Polysaccharide-related peaks were detected at 850–920 cm^−1^ (the skeleton mode of the anomeric skeletal configuration) and 1725–1880 cm^−1^ (attributed to carbonyl compounds) [14]. The peaks at 1944–2549 cm^−1^ were due to the vibration of C=C or C=O bonds of fatty acids, amide bonds of proteins, and DNA-strand bonds [9,10,11]. Broad peaks corresponding to bond vibrations of proteins or nucleic acids appeared at 2620–2766 and 3500–3600 cm^−1^ [12]. The OH–NH–CH stretching vibration afforded broad peaks at 3661–3769 cm^−1^ [7].

### 3.2. Cytotoxicity Test

**Figure 6:**
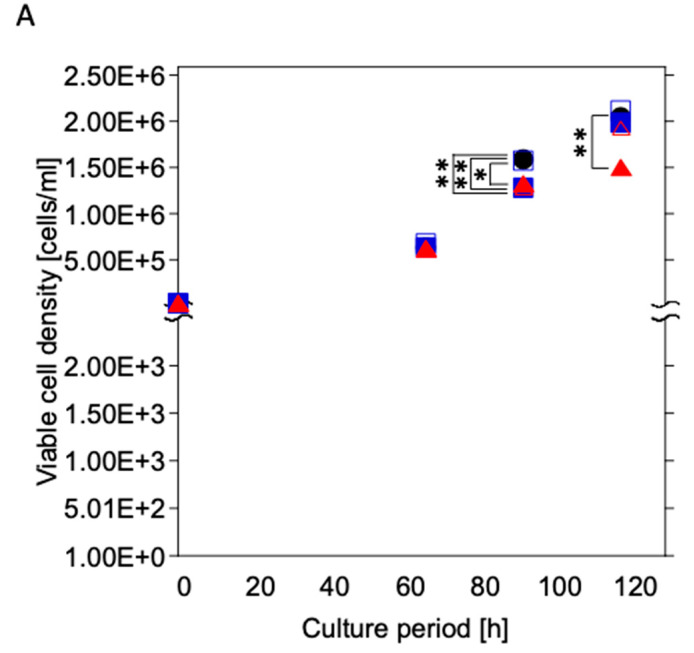

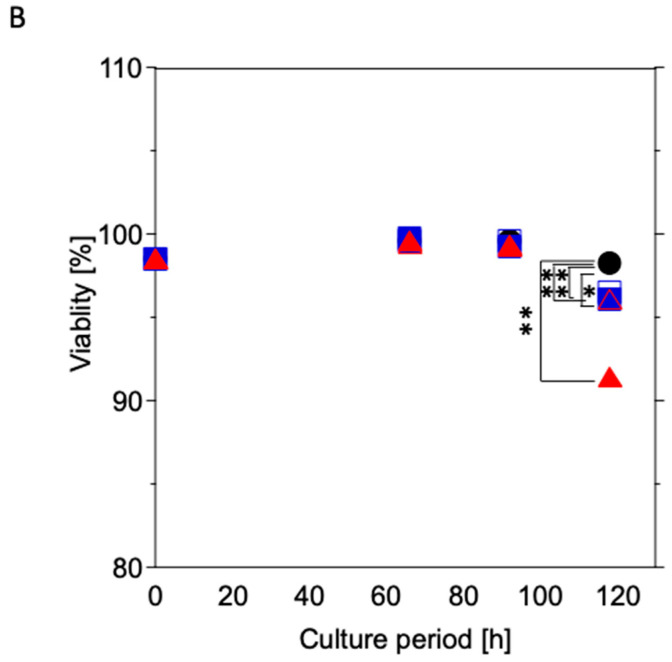
Time course of viable cell density (**A**) and viability (**B**) of U937 cultured with the test samples. Black circles: control culture (without test samples); open squares: cultured with 316L; blue squares: cultured with Coated-316L; open triangles: cultured with Ti; red triangles: cultured with Coated-Ti. Each culture condition was tested in triplicate. *: *p* < 0.05 vs. control culture condition; **: *p* < 0.01 vs. control culture condition.

The viable cell density was increased during the culture period under all culture conditions (Figure 6A). The Coated-Ti culture (U937 cells cultured with Coated-Ti) exhibited a substantially lower viable cell density than the control culture (U937 cells cultured without any test samples) at 92 h (*p* < 0.05) and 118 h (*p* < 0.01). The viable cell density of the Coated-SUS316L culture (U937 cells cultured with Coated-316L) and Ti culture (U937 cells cultured with Ti) was temporally lower than that of the control culture at 92 h (*p* < 0.01). Meanwhile, the 316L culture (U937 cells cultured with 316L) showed almost the same viable cell density as the control culture.

The viability hovered at 99% under all culture conditions until 92 h; however, it decreased at 118 h (Figure 6B). Additionally, the control culture exhibited higher viability than other culture conditions at the same culture time (Ti: *p* < 0.05 vs. control; 316L, Coated-316L, and Coated-Ti: *p* < 0.01 vs. control).

## 4. Discussion

Body-inserted biomaterials can cause chronic infectious diseases because the antibacterial function of human immune cells such as macrophages and dendritic cells is hampered by the inserted biomaterials, allowing biofilm formation on their surface. Therefore, biomaterials should exhibit antibiofilm as well as biocompatibility properties. In this study, *E. coli* and *S. epidermidis* were used as models of Gram-negative and Gram-positive biofilm-forming bacteria, respectively.

In the *E. coli* test, the four culture conditions, i.e., 316L, Ti, Coated-316L, and Coated-Ti, produced almost the same amount of biofilms. The identification of the Raman peaks was performed according to information on biological Raman spectroscopic analyses. If the position of a target Raman peak corresponded to two biological compounds, a literature search was performed to estimate the biological compounds. The Raman spectra of after-culture 316L and after-culture Ti were very similar, indicating that the components of the biofilms formed on 316L and Ti were the same and consisted mainly of polysaccharides, proteins, nucleic acids, and lipids. The largest Raman peak corresponded to a C≡N triple bond, which can be found in cyano compounds derived from the sulfur metabolism of Rhodanese enzymes in *E. coli* [18,19,20]. The OH–NH–CH bond was related to lysophosphatidic acid, which is known as an important component of biofilms [21]. The observed peaks of the siloxane-based polymer overlapped considerably with those derived from the biofilms formed on Coated-316L and Coated-Ti. Hence, we focused on particularly intense peaks to estimate the components of the biofilms. Six peaks were observed in the spectrum of after-culture Coated-316L, including that of the C≡N triple bond. Meanwhile, the spectrum of after-culture Coated-Ti displayed four intense peaks, suggesting that the composition of the biofilms formed on Coated-Ti differed from that on Coated-316L and that the sulfur metabolism of *E. coli* on Coated-Ti was weaker than that on Coated-316L.

In the case of *S. epidermidis*, the amount of biofilm formed on Coated-316L was almost the same as that on 316L and Ti. In contrast, the amount of biofilm formed on Coated-Ti was substantially higher than that on Ti. The surface of Ti contains a layer of titanium oxide. Titanium oxide is known as a photocatalyst that can kill bacteria and inhibit biofilm formation [22,23]. In this study, the biofilm formation tests were performed under fluorescent light, which would cause photocatalytic reactions on the surface of Ti. With the siloxane-based polymer coating, it was considered that the fluorescent light was partly interrupted, resulting in inhibition of the photocatalytic reaction and the progression of biofilm formation. Several peaks associated with the siloxane-based polymer were detected in after-culture Coated-316L and after-culture Coated-Ti, and these were excluded from the identification of the biofilm composition. The origin of the highest Raman peak was different among the four samples, that is, it was a lipid-derived peak for after-culture 316L, a C≡C triple-bond peak for after-culture Ti, and a cyano-derived peak for after-culture Coated-316L and after-culture Coated-Ti. Moreover, a comparison of the Raman spectra of after-culture 316L and after-culture Ti revealed that more biological compounds were detected in 316L than in Ti, even though the biofilm amount was similar in both cases. These results show that the composition of the biofilms is influenced by the surface of the basal plates.

In the cytotoxicity assay, all the test pieces (316L, Ti, Coated-316L, and Coated-Ti) exerted a negative effect on the cell survival at the end point of culture. In particular, Coated-Ti inhibited considerably the cell proliferation and cell survival in the late culture period. The viable cell concentration of Coated-316L was temporally lower than that of 316L (at 92 h). Moreover, the viable cell concentration and viability at 116 h of Coated-Ti were lower than those of Ti. These results indicate that the siloxane-based polymer coating damages cell functions such as cell survival and proliferation. This polymer was formed via the polymerization of hydrolyzed and dealcoholized methyl- and phenyl-alkoxy silane (Permiate, D & D) under humid air conditions; however, the polymerization probably did not reach completion. Therefore, the remaining alkoxyl bonds in the siloxane-based polymer coating can chemically react with the cellular membranes and polymerize, thus inhibiting normal cell proliferation and enhancing the cell death signaling pathway.

Scanning electron microscopy (SEM) is a powerful tool for elucidating the surface conditions of siloxane-based polymer coatings before and after autoclave treatment. Nevertheless, we could not obtain clear SEM images of the structure and surface conditions in this study. Therefore, we deduced the mechanism underlying the cytotoxicity of siloxane-based polymer coatings prepared by autoclave sterilizations as follows: During the sterilization of the test pieces in an autoclave, the test pieces were exposed to high pressure (2 atm) and high temperature (121 °C), which were supposed to progress the hydrothermal reactions as follows: (1) The protective oxide layers were removed from the surfaces of SUS316L and Ti, inducing leakage of metal ions from the plates. (2) Some parts of the siloxane-based polymer were disconnected in phenyl, methyl, and siloxane bonds, forming chemically active sites that can affect the survival and proliferation of U937 cells. SUS316L is mainly composed of Fe, Ni, Cr, and Mo. Fe, Ni, and Cr are prone to ionization and, hence, are easily ionized and leaked from the surface of SUS316L into the culture solution with or without a siloxane-based polymer coating. Generally, the surfaces of pure titanium materials are easily oxidized, covering the materials with a compact titanium oxide membrane. This membrane is expected to be broken during autoclave treatment, resulting in the leakage of titanium ions that affect cellular functions such as proliferation and survival. However, the leakage of metallic ions did not affect the biofilm formation by *E. coli* and *S. epidermidis* because the amount of biofilm formed on SUS316L was almost the same as that on Ti. Nevertheless, the amount of biofilm formed on Coated-Ti on *S. epidermidis* was substantially higher than that on Ti; thus, the coating could interfere with the fluorescent light reaching the surface of the Ti plate, resulting in inhibition of the photocatalytic effects (killing bacteria) of titanium. Accordingly, metallic ions can permeate the siloxane-based polymer coating, which can partially inhibit fluorescent light. Therefore, further strategies are required for the siloxane-based polymer coating to inhibit biofilm formation, such as using conjugated organic metal compounds and silver nanoparticles [24,25]. In this study, the duration of the cytotoxicity test (five days) was longer than that of the biofilm formation test (one to two days). During the first three days of the cytotoxicity test, the viable cell concentration and viability were very similar among all culture conditions, but they differed in the last two days. Hence, autoclave-related changes in the test pieces most likely appeared after more than three days of the test.

Autoclave treatment can cause the hyperthermal processing of siloxane-based polymer coatings. Therefore, siloxane-based polymer coatings are thought to be cytotoxic because of autoclave-related denaturation. The reason for the influence of siloxane-based polymer coatings on biocompatibility should be investigated in future studies. Considering the application of biomaterials, alternative sterilization methods, such as irradiation, are required to preserve the characteristics of the coatings and basal materials.

## 5. Conclusions

Inserted biomaterials can increase the risk of biofilm infections. Following our previous work on siloxane-based polymer coatings to prevent biofilm formation, in which we found that the polymer can be easily prepared from alkylalkoxysilane in humid air and coated simultaneously, and that the polymer coating was effective in inhibiting biofilm formation when it contained metal nanoparticles, graphene, and organic metal compounds, we evaluated, in this study, the biofilm formation ability and cytotoxicity of siloxane-based polymer coatings. Regardless of the siloxane-based polymer coating, the surfaces of SUS316L and Ti were activated via autoclave sterilization, resulting in the leakage of metallic ions that influenced the biofilm formation and cytotoxicity. The siloxane-based polymer coating was probably denatured during the autoclave sterilization process, affecting negatively the survival and proliferation of U937 cells but not the biofilm formation of *E. coli* or *S. epidermidis*. To apply siloxane-based polymer coatings to biomaterials, autoclave sterilization should be replaced with other sterilization methods such as UV irradiation to avoid deterioration of the coatings and leakage of metallic ions from the basal materials. In addition to sterilization, siloxane-based polymer coatings should be bioavailable, because they exhibit chemical and physical stability under marine environments (high salinity) [25] and outdoor conditions (tolerant of variable temperatures from the atmosphere of refrigerators to deserts, tolerant of sunlight) [3]. However, further enhancement of the antibiofilm activities of siloxane-based polymer coatings is required.

## Data Availability

Data available on request.
